# Reducing DNA extraction costs through factorial design for the DNAdvance Kit

**DOI:** 10.1186/s13104-024-07063-5

**Published:** 2024-12-30

**Authors:** Carson J. Neal, Zachery D. Zbinden, Michael E. Douglas, Marlis R. Douglas

**Affiliations:** 1 Department of Biological Sciences, University of Arkansas, Fayetteville, AR USA; 2 Present Address: Appalachian Laboratory, University of Maryland Center for Environmental Science, Frostburg, MD USA

**Keywords:** DNA extraction optimization, Cost-effective genotyping, DNAdvance Kit, Factorial design analysis, White-tailed Deer

## Abstract

**Objective:**

Extracting DNA is essential in wildlife genetic studies, and numerous methods are available. However, the process is costly and time-consuming for non-model organisms, including most wildlife species. Therefore, we optimized a cost-efficient protocol to extract DNA from the muscle tissue of White-tailed Deer using the DNAdvance kit (Beckman Coulter), a magnetic-bead-based approach. We devised a 3 × 3 factorial design using combinations of tissue mass (10 mg, 50 mg, or 100 mg) and reaction volume (25%, 33%, and 50% of the manufacturer's recommended volumes). DNA was extracted for *N* = 81 tissue sub-samples (9 replicates/treatment).

**Results:**

Our target yield was 500 ng of genomic DNA per sample, sufficient for population genetic assessments. A combination of 50 mg tissue and 25% reaction volume yielded enough DNA at the lowest cost. The factorial design revealed that varying tissue mass and reagent volume significantly affected extracted DNA yield. Our study demonstrates that sufficient DNA can be extracted at 75% lower costs than the manufacturer's standard protocol. Other researchers can directly use our modified DNAdvance protocol to perform cost-effective DNA extractions.

**Supplementary Information:**

The online version contains supplementary material available at 10.1186/s13104-024-07063-5.

## Background

Extracting genomic DNA or gDNA (hereafter simply DNA) from tissue samples is the first and most crucial step to genotyping—whether done for biomedical assays, population genetic studies, or human ancestry analysis. DNA extraction is the process by which nucleic acids are isolated from other cell substances, such as proteins, lipids, and carbohydrates. Once isolated, DNA is available for downstream applications, including PCR amplification or sequencing. Various DNA isolation methods exist, including organic (phenol–chloroform method), nonorganic (salting out), and adsorption (silica-gel membrane) approaches [1, 2]. However, the process can be time-consuming and unreliable in terms of yield [3, 4], depending on the tissue sources and method used [5].

DNA extraction kits accelerate and standardize DNA extraction but make it more expensive. Per-sample extraction costs can be reduced using custom protocols that use a fraction of the manufacturer's recommended reagent volumes and/or modify sample mass [2]. Our study focused on optimizing the cost-effective extraction of DNA from White-tailed Deer (*Odocoileus virginianus*) tissue using the DNAdvance Kit (Beckman Coulter, Inc.). We aimed to obtain sufficient DNA (500 ng/ sample) at the lowest cost by reducing the reagent amount and determining the minimum tissue mass.

DNAdvance is a magnetic-bead-based kit designed for high-throughput genotyping. It can be used manually or in liquid-handling robots and has been applied to extract DNA from mouse tissue [6], human cells [7], and zooplankton [8]. The kit has been evaluated against other DNA extraction methods to compare DNA yield and quality [9, 10], but none have focused on wildlife species. The protocol requires minimal training and can be performed with widely accessible equipment. First, the tissue must be lysed for about 22 h. Once visible tissue is dissolved, beads bind the nucleic acids and isolate them from the solution for ethanol washes to remove cell debris before elution of DNA and removal of beads. The process takes approximately 24 h, but the hands-on time is less than 2 h for a set of 24 samples, making it less labor-intensive than many other methods, such as silica-gel membrane techniques [11]. Much of the hands-on time is spent allocating tissues for extraction, a necessary step regardless of the extraction method.

We optimized the DNAdvance kit for muscle (tongue) tissue of White-tailed Deer as part of our recent [12, 13] and ongoing work genotyping thousands of samples to study the spread of Chronic Wasting Disease (CWD), a prion disease leading to neurological degeneration and mortality in cervids [14, 15]. CWD has been detected in 26 states across the U.S., two Canadian provinces, and three European countries [16]. Disease spread can be modeled based on patterns of gene flow among deer populations across the landscape [13, 17]. Optimizing protocols and workflows for applications in wildlife genetics will benefit efforts across a range of fields that require efficient DNA extraction, such as monitoring disease spread and containment in wildlife [18, 19].

## Main text

### Experimental design

We used a 3 × 3 factorial design, varying tissue mass of tongue (10 mg, 50 mg, 100 mg; Figure S1) and reagent volume of the DNAdvance kit (25%, 33%, and 50% of manufacturer-suggested volume). These tissue masses were selected to encompass and extend beyond the manufacturer’s recommended range (10–20 mg) to explore the potential effects of tissue mass on DNA yield. These volumes were chosen because they allow extractions to be carried out in standard 200 μL PCR strip vials (or plates). This design yielded *N* = 9 treatment combinations, and each combination was replicated *N* = 9 times for a total of *N* = 81 observations. Three replicates for each treatment combination were processed in three batches of 27 samples per week across 3 weeks.

### DNA extraction

The first step required tissue allocation for cell lysis. We used tissue from a single deer tongue to avoid the confounding effects of tissue quality variation. The tissue had been stored at − 20 °C before DNA extraction. We subdivided this tissue into three mass treatments of 10 mg, 50 mg, and 100 mg (Figure S1), with tissues allocated into 200 μL PCR strip vials (note: these vials can hold a volume of up to 300 μL without cap).

The manufacturer's standard protocol suggests a tissue mass of 20 mg and a reaction volume of 200 μL lysis master mix, containing 188 μL Lysis LBH, 5 μL 1 M DTT, and 7 μL Proteinase K (40 mg/μL) per sample (Table 1). For treatments at reagent proportions of 25%, 33%, and 50% of the recommended master mix volume, we scaled reagent amounts proportionally (Table 1). Samples were incubated at 55 °C overnight (22 h) on a GeneAmp 9700 Thermocycler (Applied Biosystems, Inc.) with a heated lid to minimize condensation.

**Table 1:** Reagent volumes (μL) for the DNAdvance kit based on the manufacturer's protocol (100%) and three adjusted volumes (25%, 33%, and 50%) were used to test differences in DNA yield.

	Percent of manufacturer's suggest volume			
	25%	33%	50%	100%
Cell Lysis				
Lysis LBH	47.0	62.0	94.0	188.0
DTT [1M]	1.3	1.7	2.5	5.0
Proteinase K [40 mg/μL]	1.8	2.3	3.5	7.0
DNA isolation				
Pre-Bind Buffer	25.0	33.0	50.0	100.0
Bind Buffer	42.5	56.1	85.0	170.0
Wash				
Ethyl Alcohol 100%	3 × 200	3 × 200	3 × 200	3 × 200
Elution				
Elution EBA Buffer 1X	30.0	30.0	30.0	30.0

Reagents are categorized into cell lysis, DNA isolation, wash, and elution. Note that the volumes for wash and elution were kept constant across all volume treatments

After tissue lysis, the standard protocol adds 100 μL of Pre-bind Buffer, followed by 170 μL of Bind Buffer containing magnetic beads (both included with the kit). For the standard protocol, this brings the total volume of a sample to 470 μL (200 μL lysis master mix + 270 μL bind buffers), which is not possible in standard 200 μL PCR strip vials or plates. For our experiment, the reagent volumes for the DNA isolation step were again scaled proportionally at 25%, 33%, and 50% of the standard protocol (Table 1). Samples were then added to a 96 M Magnum magnetic plate (Alpaqua Engineering) for 4 min to separate the DNA bound to the magnetic beads from the solution (Figure S2). The supernatant was discarded from the center of each well. The following volumes associated with DNA washing were not scaled across treatments (Table 1) since this component is not included in the extraction kit and is relatively inexpensive. Each sample was washed three times with 200 μL of 70% ethyl alcohol (not included with the kit). For elution, a consistent 30 μL of Elution EBA Buffer was used across all samples to directly compare DNA concentrations across treatments (i.e., total yield = [DNA] × 30 μL). The DNA supernatant was transferred to storage vials and conserved at − 20 °C. DNA concentration was measured using the Qubit Broad-Range Assay on a Qubit 2.0 Fluorometer (Invitrogen, Inc.) [20, 21].

### Data analysis

We compared DNA concentration among treatment groups using Analysis of Variance (ANOVA) followed by Tukey's Honestly Significant Difference (HSD) test for multiple comparisons. ANOVA has been used elsewhere to evaluate DNA extraction methods [22–24]. DNA concentration is reported with yield implied, given that total elution volume was constant across treatments (30 μL).

Initial analyses showed that residuals significantly deviated from normality based on Shapiro–Wilk and Kolmogorov–Smirnov tests, prompting a log transformation of DNA concentrations that solved this issue. The overall results were unchanged between analyses on transformed and untransformed data (not shown). To confirm the absence of batch effects, we conducted a one-way ANOVA on log-transformed DNA concentrations across processing batches (weeks) and found no significant effect.

To evaluate the effects of tissue mass and reagent volume on DNA concentration and yield, we applied a two-way ANOVA. Post-transformation, we confirmed residual normality using the Shapiro–Wilk and Kolmogorov–Smirnov tests. While ANOVA identifies overall differences among groups, it does not specify which pairs differ. Therefore, Tukey’s HSD test was used for post hoc comparisons to determine statistical differences between specific treatment combinations after applying the Tukey–Kramer method to adjust *p*-values due to multiple comparisons and control the family-wise error rate (FWER). Tukey’s HSD is frequently used to compare DNA extraction protocols [4, 25–27]. All analyses were conducted using GPT-4o (OpenAI) and independently confirmed using base R statistical programming [28]. The complete data set is provided in the supplement (Table S1).

### Results

**Fig. 1:**
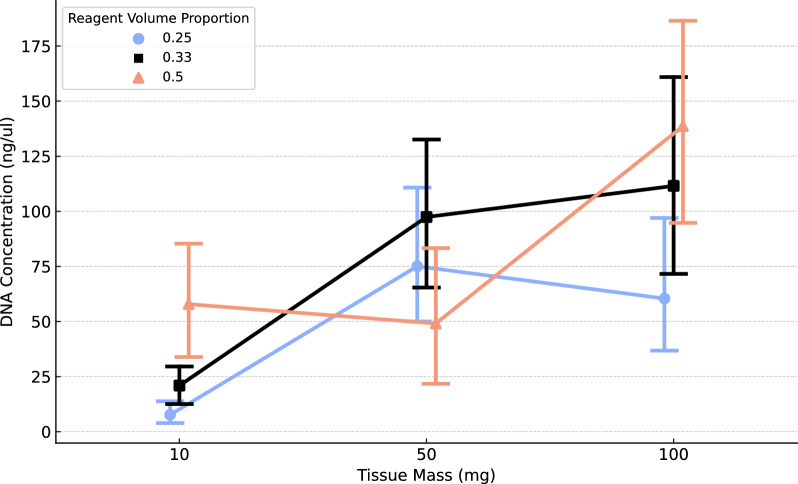
Interaction effect of tissue mass and reagent volume proportion on DNA concentration. Mean DNA concentrations (ng/µL) are shown for each combination of tissue mass (mg) and reagent volume (as a percentage of the manufacturer’s suggested total volume). Error bars show the standard error of the mean

The ANOVA indicated no batch effects across weeks due to the balanced design (*F*_2,78_ = 0.98, *p* = 0.379). DNA concentrations differed significantly among treatments (Table 2, Fig. 1). Both tissue mass (*F*_2,72_ = 35.85, *p* = 1.57 × 10^–11^) and reagent volume (*F*_2,72_ = 8.01, *p* = 7.2 × 10^–4^) significantly affected DNA concentration. However, the interaction between tissue mass and reagent volume was also significant (*F*_2,72_ = 8.57, p = 1.03 × 10^–5^), indicating that certain combinations of mass and volume yield markedly different DNA concentrations.

**Table 2:** We devised a 3 × 3 factorial design using combinations of tissue mass (10 mg, 50 mg, or 100 mg) and reaction volume (25%, 33%, and 50% of the DNAdvance manufacturer's recommended volumes) as variables to create nine treatment combinations and extracted DNA for 81 samples (9 replicates/treatment)

Mass (mg)	Volume (proportion)	Mean [DNA] (ng/μL)	Mean log [DNA]	Standard Error log[DNA]
10	25%	7.65	1.70	0.25
10	33%	20.92	2.72	0.34
10	50%	57.88	3.82	0.25
50	25%	75.04	4.16	0.19
50	33%	97.42	4.41	0.22
50	50%	49.09	3.44	0.35
100	25%	60.42	3.90	0.21
100	33%	111.52	4.55	0.20
100	50%	138.63	4.80	0.19

The extracted DNA concentration increased with greater tissue mass (Fig. 1), although the interaction with reagent volume precludes a straightforward interpretation of the main effects. We report average main effect trends for context. Compared to the minimum tissue mass of 10 mg (x̄ = 28.82 ± 32.91 ng/μL), the mean DNA concentration increased by about 156% for 50 mg of tissue mass (x̄ = 73.85 ± 52.51 ng/μL) and 259% for 100 mg (x̄ = 103.53 ± 72.41 ng/μL). The log-transformed DNA concentrations for the two larger masses differed significantly from the smallest mass treatment (Table 3).

**Table 3:** Post hoc analysis of three tissue mass levels and their mean DNA concentrations after extraction

Mass compared (mg)	Mean difference [DNA] (ng/μL)	Percent difference [DNA]	Adjusted *p*-value	Significant difference
10 vs 50	45.04	156%	< 0.001	Yes
10 vs 100	74.71	259%	< 0.001	Yes
50 vs 100	29.67	40%	0.259	No

The percent difference between concentrations was calculated relative to the smaller concentration: % Diff. = (Diff / Lowest value) × 100. The analysis was performed on log-transformed DNA concentrations, but differences in actual values are shown for a more straightforward interpretation. The *p*-values were adjusted using the Tukey–Kramer method

Compared to the minimum reagent volume of 25% (x̄ = 47.70 ± 48.73 ng/μL), mean DNA concentration increased by 61% for a volume proportion of 33% (x̄ = 76.62 ± 64.80 ng/μL) and 72% for a volume proportion of 50% (x̄ = 81.87 ± 68.88 ng/μL). However, only one post hoc comparison (25% vs. 50%) was statistically significant (Table 4). Therefore, the volume effect likely depends on mass, as evinced by the significant interaction between the two noted above.

**Table 4:** Post hoc analysis of three reagent volume levels and their mean DNA concentrations after extraction

Volume compared	Mean difference [DNA] (ng/μL)	Percent difference [DNA]	Adjusted *p*-value	Significant difference
25 vs 33%	28.92	61%	0.104	No
25 vs 50%	34.16	72%	0.041	Yes
33 vs 50%	5.24	7%	0.914	No

The percent difference between concentrations was calculated relative to the smaller concentration: % Diff. = (Diff/Lowest value) × 100. The analysis was conducted on log-transformed DNA concentrations; however, differences in actual values are presented for easier interpretation. The *p*-values were adjusted using the Tukey–Kramer method

Figure 1 and Table 2 indicate that mass and reagent volume were positively associated with DNA concentration/yield. However, the increases in DNA concentration were only significant between 25 and 50% reagent volume (Table 4). The mean difference in DNA concentration between 33 and 50% reagent volume was minuscule and non-significant. Given the yield of > 500 ng (20 ng/μL × 30 μL) is consistently achieved with 50 mg of tissue at 25% reagent volume, this is an optimal combination to reduce extraction-associated costs. Using the manufacturer’s standard protocol, the reagent cost per sample is $3.21 USD (based on $1,234 for 384 samples at the time of this writing). In contrast, our optimized protocol reduces this reagent cost to $0.80 per sample. This estimate excludes user-supplied consumables (e.g., gloves, pipette tips, vials, and ethyl alcohol).

## Limitations

Our study demonstrated a cost-efficient approach to obtaining sufficient amounts of genomic DNA from a wildlife species using a scaled-down, customized protocol of the DNAdvance Kit. One caveat to scaled-down reagent volumes is that pipetting errors are proportionally magnified and thus more prone to human error. Automated liquid handling solutions would minimize inconsistent dispense volumes and reduce this source of error.

Another limitation involves the exclusive use of tongue from White-tailed Deer in the experiment, which may not translate directly to other tissue types or species [18, 19]. Furthermore, we opted to use sub-samples from a single tissue source from a single individual to remove any effect of tissue quality variation from our experiment. In practice, sample quality may vary and influence DNA extraction yield.

Finally, our experiment did not include a treatment using the full volume (100%) of the manufacturer’s recommended reagents, which could have served as a baseline. Our goal was to scale down the volumes to enable extractions in standard 200 μL PCR strip vials (not possible with full volume); this limited the maximum volume and prevented direct comparisons without introducing potential confounding factors, such as vial type.

## Discussion

This study optimized the DNAdvance Kit for cost-effective genomic DNA extraction from a non-model mammal species, White-tailed Deer. We demonstrated that manipulating sample tissue mass and reagent volume significantly affected DNA yield and concentration. Most DNA extraction evaluations focus on the similarities and differences between methods or commercial kits [3, 4, 29]. Instead, we focused on optimizing a single kit to minimize per-sample costs while consistently obtaining a minimum yield of 500 ng DNA. Our target yield is sufficient for the conservation genomic applications that rely on Sanger [12] and next-generation [13] sequencing. DNA yield requirements can vary significantly depending on the genomic application. For example, standard PCR and basic genotyping may require as little as 1–100 ng [11]. In contrast, more data-intensive applications like whole-genome sequencing typically require upwards of 1000 ng of high-quality DNA, although magnetic bead-based protocols, such as those used here, have demonstrated that as little as 25 ng may be sufficient [29].

Based on our results, we conclude that DNA yield using the extraction kit is influenced by both tissue mass and reagent volume, with evidence for a significant interaction between these factors (Fig. 1). This interaction limits the interpretation of main effects in isolation; thus, we cannot definitively attribute differences in mean DNA concentrations to specific reagent volume levels (e.g., 25% vs. 33%, 25% vs. 50%, or 33% vs. 50%). Therefore, the observed main effect of reagent volume may be influenced by the interactive effect with tissue mass rather than reflecting consistent differences across reagent volumes alone.

Generally, DNA yield increased with both tissue mass and reagent volume. For reagent volumes, the rank of DNA yield was 50%, 33%, and 25% (highest to lowest) at 10 mg and 100 mg tissue masses (Fig. 1). However, a discrepancy was noted for the 50 mg tissue mass, where the 50% reagent volume yielded the lowest DNA concentration (Fig. 1). The consistency of the pattern at 100 mg tissue mass suggests that neither reagent saturation nor tissue mass limitations likely account for the anomaly observed at 50 mg tissue mass. Instead, this deviation may reflect specific variability in extraction efficiency at this intermediate mass, potentially due to subtle process dynamics. Further investigation into DNA yield at intermediate mass and volume combinations could provide additional clarity.

Additionally, the effect of reagent volume was more distinct at 10 mg tissue mass, with more apparent separation among volume groups (Fig. 1). In contrast, the 50 mg and 100 mg tissue mass treatments showed greater variability, as indicated by larger standard errors (and treatment overlap), and a less consistent increase in DNA concentration with higher reagent volumes (Fig. 1). The higher DNA concentration at 50 mg tissue mass X 25% reagent volume compared to 100 mg X 25% may reflect differences in reagent efficiency across tissue masses. These observations suggest optimal reagent volumes could vary with tissue mass, supporting further protocol customization for different sample types.

The importance of these results lies in their generalizability—DNA extraction is used in numerous applications across several different fields, including paleontology [30], biomedical research [31], and forensic sciences [32]. No published study has been conducted on optimizing the DNAdvance Kit, so this data can be helpful to any researcher considering this approach.

This study can have additional implications beyond cost reduction. DNA extraction and subsequent genotyping are crucial in studying disease transmission, such as chronic wasting disease (CWD) [12, 13]. CWD is an urgent concern for wildlife conservation due to its impact on cervid populations [16]. Our data provide a valuable resource to researchers aiming to understand the spread of CWD. These results can also highlight the importance of experiment-specific alteration of DNA extraction protocols [18, 19]. Our focus on the cost-effectiveness of the DNAdvance kit provides a novel contribution to the research community.

## Conclusions

In conclusion, this study successfully demonstrates a cost-effective approach to DNA extraction using the DNAdvance kit, achieving significant reductions in reagent use while yielding sufficient DNA for most downstream applications. We found a combination of 50 mg tissue and 25% reaction volume yielded enough DNA at the lowest cost. This optimization presents valuable insight, especially for resource-limited labs. Moreover, the factorial design outlined in this study provides a simple framework that can be adapted and refined for various biological tissues and species. Ultimately, this research underscores the importance of protocol customization to optimize genetic studies involving non-model organisms such as fish and wildlife, paving the way for more sustainable and accessible scientific exploration.

## Supplementary Information

Supplementary Material 1.

## Data Availability

All data are available in the Supplementary Material.

## Abbreviations

gDNA: Genomic DNA
mg: Milligram
ng: Nanogram
μL: Microliter
AR: Arkansas
CWD: Chronic wasting disease
ANOVA: Analysis of variance
HSD: Honestly significant difference
DTT: Dithiothreitol
PCR: Polymerase chain reaction
LBH: Lysis buffer high
EBA: Elution Buffer A
DNA: Deoxyribonucleic acid
USD: United States Dollar
