# Supplementary material for: Reducing DNA extraction costs through factorial design for the DNAdvance Kit
Source: BMC Res Notes. 2024 Dec 30;17:397. doi: 10.1186/s13104-024-07063-5 (PMC11684086; doi:10.1186/s13104-024-07063-5)
Supplement: Supplementary file 1 — Supplementary Material 1. [file 13104_2024_7063_MOESM1_ESM.docx]

**SUPPLEMENTARY MATERIAL**

**Reducing DNA Extraction Costs Through Factorial Design for the DNAdvance Kit**

Carson J. Neal^1^ (carsonn@uark.edu),

Zachery D. Zbinden^1,2*^ (zach.zbinden@umces.edu),

Michael E. Douglas^1^ (med1@uark.edu),

Marlis R. Douglas^1^ (mrd1@uark.edu)

^1^Department of Biological Sciences, University of Arkansas, Fayetteville, AR, USA

^2^Current address: Appalachian Laboratory, University of Maryland Center for Environmental Science, Frostburg, MD, USA

*Corresponding author: Zachery D. Zbinden

**
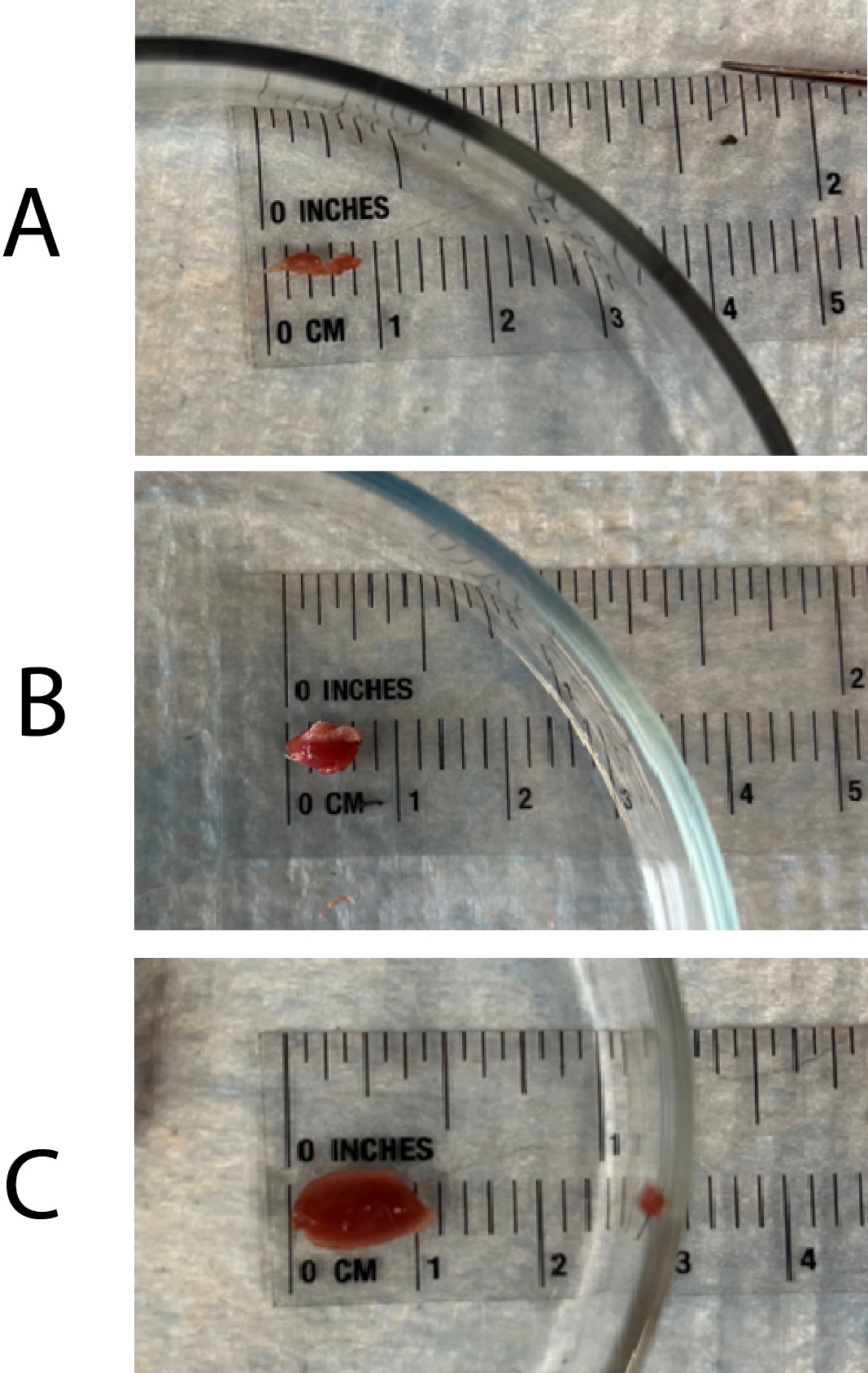
**

**Figure S1:** Three masses of White-tailed Deer tongue tissue from which DNA was extracted: A) 10mg, B) 50mg, and C) 100mg.


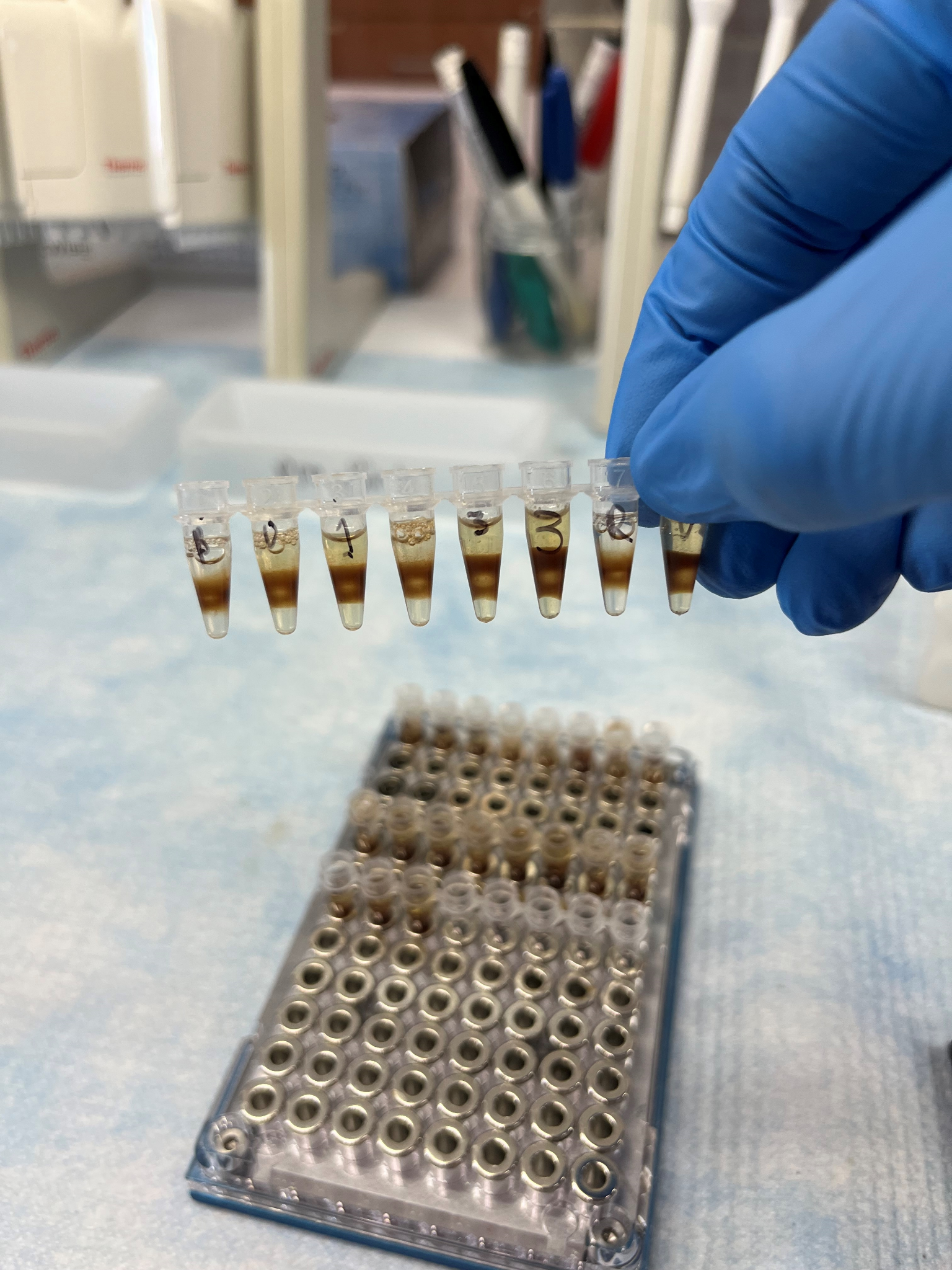


**Figure S2:** DNA bound to magnetic beads, which are attached to the sides of the tubes. During this step, the slightly yellow supernatant was removed without disturbing beads on the side, ideally leaving only DNA and magnetic beads.

**Table S1:** DNA concentration data collected for N=81 samples of White-tail Deer tissue across N=9 experimental treatment groups and three weekly blocks. Sample ID is a unique identifier for each sample. The week represents the weekly block in which the samples were extracted. The mass of the tissue is given in grams, and the volume of DNAdvance kit lysis reagent used is given as the percentage of the manufacturer's protocol. The final DNA Concentration is given in ng/μl.

| **Sample ID** | **Week** | **Mass (g)** | **Volume (%)** | **DNA Concentration (ng/μl)** |
| --- | --- | --- | --- | --- |
| B01V25M10A | 1 | 10 | 0.25 | 3.93 |
| B01V25M10B | 1 | 10 | 0.25 | 3.22 |
| B01V25M10C | 1 | 10 | 0.25 | 8 |
| B02V25M10D | 2 | 10 | 0.25 | 2.45 |
| B02V25M10E | 2 | 10 | 0.25 | 3.31 |
| B02V25M10F | 2 | 10 | 0.25 | 6.42 |
| B03V25M10G | 3 | 10 | 0.25 | 30.7 |
| B03V25M10H | 3 | 10 | 0.25 | 4.26 |
| B03V25M10I | 3 | 10 | 0.25 | 6.53 |
| B01V25M50A | 1 | 50 | 0.25 | 65.9 |
| B01V25M50B | 1 | 50 | 0.25 | 192 |
| B01V25M50C | 1 | 50 | 0.25 | 102 |
| B02V25M50D | 2 | 50 | 0.25 | 25.8 |
| B02V25M50E | 2 | 50 | 0.25 | 33.4 |
| B02V25M50F | 2 | 50 | 0.25 | 68.9 |
| B03V25M50G | 3 | 50 | 0.25 | 62 |
| B03V25M50H | 3 | 50 | 0.25 | 62.6 |
| B03V25M50I | 3 | 50 | 0.25 | 62.8 |
| B01V25M100A | 1 | 100 | 0.25 | 183 |
| B01V25M100B | 1 | 100 | 0.25 | 68.1 |
| B01V25M100C | 1 | 100 | 0.25 | 74.8 |
| B02V25M100D | 2 | 100 | 0.25 | 34.6 |
| B02V25M100E | 2 | 100 | 0.25 | 49.3 |
| B02V25M100F | 2 | 100 | 0.25 | 21.6 |
| B03V25M100G | 3 | 100 | 0.25 | 35.6 |
| B03V25M100H | 3 | 100 | 0.25 | 47.5 |
| B03V25M100I | 3 | 100 | 0.25 | 29.3 |
| B01V33M10A | 1 | 10 | 0.33 | 47.8 |
| B01V33M10B | 1 | 10 | 0.33 | 17.6 |
| B01V33M10C | 1 | 10 | 0.33 | 19.1 |
| B02V33M10D | 2 | 10 | 0.33 | 35.4 |
| B02V33M10E | 2 | 10 | 0.33 | 19.2 |
| B02V33M10F | 2 | 10 | 0.33 | 16.7 |
| B03V33M10G | 3 | 10 | 0.33 | 24.7 |
| B03V33M10H | 3 | 10 | 0.33 | 1.56 |
| B03V33M10I | 3 | 10 | 0.33 | 6.24 |
| B01V33M50A | 1 | 50 | 0.33 | 189 |
| B01V33M50B | 1 | 50 | 0.33 | 63.3 |
| B01V33M50C | 1 | 50 | 0.33 | 132 |
| B02V33M50D | 2 | 50 | 0.33 | 44.1 |
| B02V33M50E | 2 | 50 | 0.33 | 62 |
| B02V33M50F | 2 | 50 | 0.33 | 92.6 |
| B03V33M50G | 3 | 50 | 0.33 | 115 |
| B03V33M50H | 3 | 50 | 0.33 | 24.8 |
| B03V33M50I | 3 | 50 | 0.33 | 154 |
| B01V33M100A | 1 | 100 | 0.33 | 91.3 |
| B01V33M100B | 1 | 100 | 0.33 | 163 |
| B01V33M100C | 1 | 100 | 0.33 | 143 |
| B02V33M100D | 2 | 100 | 0.33 | 97.7 |
| B02V33M100E | 2 | 100 | 0.33 | 50.7 |
| B02V33M100F | 2 | 100 | 0.33 | 38 |
| B03V33M100G | 3 | 100 | 0.33 | 269 |
| B03V33M100H | 3 | 100 | 0.33 | 60.7 |
| B03V33M100I | 3 | 100 | 0.33 | 90.3 |
| B01V50M10A | 1 | 10 | 0.5 | 85 |
| B01V50M10B | 1 | 10 | 0.5 | 48.6 |
| B01V50M10C | 1 | 10 | 0.5 | 33.4 |
| B02V50M10D | 2 | 10 | 0.5 | 136 |
| B02V50M10E | 2 | 10 | 0.5 | 102 |
| B02V50M10F | 2 | 10 | 0.5 | 25.9 |
| B03V50M10G | 3 | 10 | 0.5 | 55 |
| B03V50M10H | 3 | 10 | 0.5 | 17 |
| B03V50M10I | 3 | 10 | 0.5 | 18 |
| B01V50M50A | 1 | 50 | 0.5 | 6.3 |
| B01V50M50B | 1 | 50 | 0.5 | 63.9 |
| B01V50M50C | 1 | 50 | 0.5 | 24.9 |
| B02V50M50D | 2 | 50 | 0.5 | 10.4 |
| B02V50M50E | 2 | 50 | 0.5 | 142 |
| B02V50M50F | 2 | 50 | 0.5 | 35.6 |
| B03V50M50G | 3 | 50 | 0.5 | 16.1 |
| B03V50M50H | 3 | 50 | 0.5 | 112 |
| B03V50M50I | 3 | 50 | 0.5 | 30.6 |
| B01V50M100A | 1 | 100 | 0.5 | 172 |
| B01V50M100B | 1 | 100 | 0.5 | 124 |
| B01V50M100C | 1 | 100 | 0.5 | 239 |
| B02V50M100D | 2 | 100 | 0.5 | 91.7 |
| B02V50M100E | 2 | 100 | 0.5 | 117 |
| B02V50M100F | 2 | 100 | 0.5 | 64.5 |
| B03V50M100G | 3 | 100 | 0.5 | 51.5 |
| B03V50M100H | 3 | 100 | 0.5 | 110 |
| B03V50M100I | 3 | 100 | 0.5 | 278 |
